# Impact of Inhibitors and L2 Antibodies upon the Infectivity of Diverse Alpha and Beta Human Papillomavirus Types

**DOI:** 10.1371/journal.pone.0097232

**Published:** 2014-05-09

**Authors:** Kihyuck Kwak, Rosie Jiang, Joshua W. Wang, Subhashini Jagu, Reinhard Kirnbauer, Richard B. S. Roden

**Affiliations:** 1 Department of Pathology, The Johns Hopkins University, Baltimore, Maryland, United States of America; 2 Department of Gynecology and Obstetrics, The Johns Hopkins University, Baltimore, Maryland, United States of America; 3 Department of Oncology, The Johns Hopkins University, Baltimore, Maryland, United States of America; 4 Laboratory of Viral Oncology, Division of Immunology, Allergy and Infectious Diseases, Department of Dermatology, Medical University Vienna (MUW), Vienna, Austria; National Institute of Health - National Cancer Institute, United States of America

## Abstract

The licensed human papillomavirus (HPV) vaccines elicit type-restricted immunity but do not target cutaneous HPV types of the beta genus that are associated with non-melanoma skin cancer in immune-compromised patients, and it is unclear if these diverse types share a common mechanism of infection. Residues 11-88 of minor capsid protein L2 contain cross-protective epitopes, and vaccination with concatamers of this region derived from as many as eight alpha HPV (L2 α11-88x8) is being developed as an alternative prophylactic vaccine with potentially broader efficacy. There is also interest in developing broadly protective topical microbicides, such as carrageenan or heparin that block HPV receptor interactions, or small molecule inhibitors of infection. Here we have examined several inhibitors of HPV infection and antisera to L2 α11-88x8 for their breadth of activity against infection by 34 HPV types from within both the alpha and beta families using pseudovirions (PsV) carrying a luciferase reporter as surrogates for native virus. We observed that both heparin and carrageenan prevented infection by mucosatropic HPV types, but surprisingly PsV of several epidermotropic alpha4 and beta HPV types exhibited increased infectivity especially at low inhibitor concentrations. Furin and γ-secretase inhibitors and L2 α11-88x8 antiserum blocked infection by all HPV PsV types tested. These findings suggest that the distinct tropism of mucosal and cutaneous HPV may reflect distinct cell surface receptor interactions, but a common uptake mechanism dependent upon furin and γ-secretase proteolytic activities. Carrageenan, which is being tested as a vaginal microbicide, broadly inhibited infection by the high-risk mucosatropic HPV PsV, but not most skin tropic alpha and beta HPV. Vaccination with an L2 multimer derived exclusively from alpha papillomavirus sequences induced antibodies that broadly neutralized PsV of all 34 HPVs from within both the alpha and beta families, suggesting each displays conserved L2 neutralizing epitopes.

## Introduction

Human papillomaviruses (HPV) comprise a family of at least 120 non-enveloped epitheliotropic viruses which contain a double-stranded circular DNA genome and are phylogenetically classified into five genera; alpha, beta, gamma, mu and nu [1]. Papillomavirus infections generally produce benign papillomas or warts of either skin or mucosa, such as condylomata accuminata (anogenital warts) associated with ‘low-risk’ types HPV6 and 11. However, the sexually transmitted ‘high-risk’ members of the alpha genus mucosal HPVs are essential etiological agents in cervical cancer, and also in a significant fraction of anal, penile, vaginal, vulval and oropharyngeal cancers [2], [3]. HPV16 and HPV18 are the most impactful high-risk HPV types, together causing 70% of cervical cancer, with a dozen or so other alpha HPVs associated with the remaining cases [4], [5], although it is important to recognize that the majority of infections are cleared by patients. The beta HPVs infect skin beginning early in childhood and are associated with non-melanoma skin cancer in sun-exposed areas of immunocompromised patients or those with the rare hereditary disease epidermodysplasia verruciformis (EDV), notably HPV5 and HPV8 [6]. Conversely, beta HPV infections are generally clinically inapparent in immune competent patients, but may cooperate with UV-induced DNA damage in the development of cutaneous squamous cell cancers [6]. Infections with HPV of the gamma, mu and nu genera typically produce benign and self-limiting skin warts [1].

Papillomavirus virions have a non-enveloped 60 nm diameter capsid with T = 7*d* iscosahedral symmetry [7]. The capsid is formed from 360 molecules of the major capsid protein L1 via assembly of 72 star-shaped capsomers or pentamers, each comprising five L1 molecules. The capsid also contains as many as 72 molecules of the minor capsid protein L2 and, while its location is not totally clear, at least a portion of L2 is buried at the base of central cavity at the center of each capsomer [8]. Five surface loops of L1 with high variation in amino acid sequence among different types contain the immunodominant neutralization epitopes and act as domain linkers for the conserved internal jelly roll structure [9].

Recombinant expression of L1 is sufficient to form virus-like particles (VLP) that mimic native virus morphologically and immunologically. Similar to infectious virions, VLP can bind to heparan sulfate proteoglycans (HSPG) on the cell surface, and HSPG mimetics such as soluble heparan sulphate or carrageenan compete this interaction [10], [11], [12], [13], [14], [15], [16]. Indeed, consistent use of the carrageenan-based vaginal microbicide, Carraguard, with condoms was negatively associated with the acquisition of high-risk HPV infections in a randomized, double-blind and placebo controlled trial [17]. L2 facilitates viral genome encapsidation during virion assembly and is critical for infection [18], [19]. At the initiation of infection, virions undergo a conformational change that reveals the amino terminus of L2 on the capsid surface such that it is cleaved by the cellular protease furin [20], [21]. Furin cuts L2 at residue 11 in HPV16 and this cleavage is required for infection [20]. L2 interacts with several cellular components, including cyclophilin, annexin, syntaxin-18 and sorting nexin 17, believed to contribute to infectious cell entry, although the mechanisms are not fully elucidated [22], [23], [24], [25]. L2 also enables virion egress from the endosome and translocates with the viral genome to the nucleus to establish infection [26]. The regulated intra-membrane protease γ-secretase is required for endosomal escape, but its cellular target remains to be defined [27], [28].

The licensed HPV vaccines, Cervarix and Gardasil, were both developed to prevent HPV16- and HPV18-associated persistent anogenital infection and disease, in particular epithelial dysplasias and cancers, and Gardasil to also prevent benign genital warts associated with HPV6 and HPV11 [29]. Vaccination elicits type-restricted neutralizing antibodies and protection due to immunodominant epitopes formed by hyper-variable surface loops of the L1 VLP antigens [9]. Both vaccines provide limited cross-protection, particularly toward HPV31 or HPV45, close phylogenetic relatives ofHPV16 and HPV18, respectively [30], [31], [32]. However the duration of cross-protection is unclear, and is weak to ineffective for more distantly related types [33]. A nonavalent L1 VLP vaccine is currently in development to broaden protection among the high-risk mucosal alpha HPV types (http://clinicaltrials.gov/show/NCT00543543). Another approach is vaccination with the amino terminus of L2 including residues 11-88 [34], [35], [36]. This region of L2 is well conserved and can elicit broadly neutralizing antibodies, although at lower titer than L1 VLP, because L2 does not form an immunogenic VLP structure. However, because L2 protective epioptes are linear, those derived from multiple HPV types can be concatenated into a single antigen, e.g. α11-88x8 that compromises a fusion of the 11-88 region of the eight medically significant alpha HPV types 6, 16, 18, 31, 39, 51, 56 and 73. Vaccination with L2 α11-88x8, or passive transfer of its antiserum, provides broad immunity against vaginal challenge with alpha HPV, but its activity against other genera has not been examined [35].

Our mechanistic understanding of papillomavirus infectious process(es) is based on studies generally performed with only a few HPV types. Here we examine if medically significant HPV types of different genera, carcinogenic potential and tropism share a common infection mechanism. As the known inhibitors and vaccines have also been tested predominantly against a limited number of HPV types, their breadth of activity cannot be fully appreciated. Further, several studies indicate differences in receptor usage and uptake pathways by different HPV types [14], [37], [38]. Because of the technical difficulties of culturing and measuring the infectivity of papillomaviruses *in vitro*, PsV are used as surrogates for biologic studies of native virions, particularly given their capacity to deliver a reporter construct upon its encapsidation by over-expression of L1 and L2 [39]. In light of this, we generated PsV for 34 HPV types from the alpha and beta genera to better understand the commonalities and differences in the infectious pathway of diverse HPV types with distinct biology.

## Materials and Methods

### Pseudovirion (PsV) production

L1/L2 expression plasmids were provided by Martin Müller and Lutz Gissmann (DKFZ, Heidelberg, Germany), Christopher Buck and John Schiller (NCI, Bethesda, MD), and Helena Faust (Lund University, Malmö, Sweden) and Joakim Dillner (Karolinska Institutet, Stockholm, Sweden). Additional constructs were synthesized by Biobasic (Table S1). Briefly, the L1 and L2 genes for individual isolates of HPV genotypes documented in Genbank were codon-optimized for expression in human cells and cloned with a 5′ Kozak sequence (GCCACC) and two 3′ stop codons (TAATAG) into the mammalian double expression vector pVITRO1-neo-mcs (InvivoGen) as listed in Table S1. The L1 gene flanked by BamHI-XbaI was cloned into the BglII-NheI region of MCS2, followed by the L2 gene flanked by BamHI-XbaI which was cloned into the BamHI-AvrII region of MCS1. All HPV PsV were produced as described in http://home.ccr.cancer.gov/Lco/pseudovirusproduction.htm. Briefly, plasmids containing L1 and L2 late genes of different HPV types were transfected with plasmid expressing luciferase reporter gene into 293TT cells utilizing TransIT-2020 transfection reagent. Cells were incubated for 48 h at 37°C, harvested, washed with DPBS-Mg (DPBS supplemented with 9.5 mM MgCl_2_), and lysed with equal volume of lysis buffer (DPBS supplemented with 0.5% w/v Brij58 and 0.2% v/v benzonase). For PsV maturation, cell lysates were incubated for 24 h at 37°C and adjusted to 850 mM NaCl. For extraction lysates were centrifuged at 10,000×*g* for 10 min, and clarified supernatants loaded onto a 12 mL step gradient of Optiprep (39, 33, 27% *w/v*), and spun at 40,000 rpm in a SW40 rotor for 15 h at 16°C. After centrifugation, 1 mL fractions were collected from the top layer and the fraction demonstrating the highest infectivity was selected for further experiments.

### HPV Infectivity assays

1.5×10^4^ 293TT, HeLaT or HaCaT cells in 100 µL DMEM-10 or KH-SV cells in 100 µL KSFM were infected with 250, 500, 1000, or 2000 million particles of each HPV types in 100 µL medium in triplicate and incubated for 72 h at 37°C. Luciferase activity was measured as described in inhibition assays. All experiments were performed in triplicate.

### HPV Inhibition assays

293TT cells were seeded at 1.5×10^4^ cells in 100 µL 10% FBS DMEM (DMEM-10) per well in 96-well plates and allowed to attach overnight. For heparin and carrageenan inhibition studies, heparin (Sigma H-4784) and carrageenan (Sigma C1138) were serially diluted in DMEM-10 and incubated with 250 million PsV particles of different HPV types in 100 µL DMEM-10 for 1 h at 37°C. Mixtures were added to 293TT cells and incubated at concentrations of 64, 128, 256, 512 µg/mL of heparin or carrageenan for 72 h at 37°C. For furin inhibition studies, furin inhibitor 1 (Decanoyl-RVKR-CMK) was mixed with 250 million particles of each HPV type in 100 µL DMEM-10 and the mixtures were immediately transferred to 293TT cells and incubated at 20 µM concentration of furin inhibitor 1 for 72 h at 37°C. For γ-secretase inhibition studies, XXI was mixed with 250 million particles of each HPV type in 100 µL DMEM-10 and the mixtures were immediately transferred to 293TT cells and incubated at a final concentration of 500 nM XXI for 72 h at 37°C. As a positive control, 4 µL of L1 antiserum from mice vaccinated i.m. with L1 DNA utilizing electroporation in 50 µL of culture medium was mixed with 250 million particles of each HPV type in 50 µL. Mixtures were transferred to 293TT cells and incubated for 72 h at 37°C. Following infection cells were washed with 1× PBS, and lysed with 30 µL of Cell Culture Lysis Reagent on rocking shaker for 15 min at room temperature. Lysates were transferred to 96-well black plate and luciferase activity was measured by GloMax-Multi Detection System after adding 50 µL of luciferin substrate to each well. All experiments were performed in triplicate.

### Neutralization assay

293TT cells were pre-plated at 1.5×10^4^ cells per well in 96-well plate and incubated 24 h at 37°C. 4 µL of rabbit L2 α11-88x8 antiserum [35] was serially diluted two-fold in 50 µL of culture medium. As a negative control, pre-immune serum was applied. 250 million particles of each HPV type in 50 µL of culture medium were mixed with serially diluted serum in triplicate and then transferred to pre-plated 293TT cells and cultured for 72 h at 37°C. Luciferase activity was measured as described above.

### SDS-PAGE and Western blotting

Each HPV PsV preparation was normalized by the amount of L1 protein. Samples were boiled for 5 min with reducing gel sample buffer, subjected to SDS-PAGE analysis using 4-20% Tris-HCl gels, and proteins were transferred to PVDF membrane. The membrane was blocked with 5% non-fat milk in PBST (PBS containing 0.1% Tween-20) at room temperature for 1 hour, incubated with a rabbit L2 α11-88x8 antiserum at room temperature for 1 hour and binding detected with HRP-conjugated goat anti-rabbit IgG secondary antiserum.

### Quantitative real time PCR measurement of luciferase reporter plasmid

Reporter plasmid was extracted from 20 µL of each HPV PsV preparation using PureLink Viral RNA/DNA extraction kits. The real time PCR was performed with 25 µL of universal PCR Master Mix, 900 nM of forward and reverse primers (TTG ACC GCC TGA AGT CTC TGA and ACA CCT GCG TCG AAG ATG TTG), 250 nM of TaqMan probe (6FAM-CCG CTG AAT TGG AAT CCA TCT TGC TC-TAMRA), and 5 µL of extracted reporter plasmid in 50 µL final volume using an I-Cycler IQ (Bio-Rad). Each sample was conducted in duplicate.

### Data analysis and statistics

To calculate the neutralization titer value (the reciprocal of the dilution that causes 50% reduction in luciferase activity), the non-linear model Y = Bottom + (Top-Bottom) /(1+10∧((LogEC50-X)*HillSlope)) was fitted to the log_10_ transformed neutralization data using Graphpad Prism 6 and the estimated EC50 [with 95% confidence intervals] is reported as the titer. Comparisons between the infectivity of members of papillomavirus species was determined by the Mann Whitney test, and means ± standard error are presented graphically using Graphpad Prism 6.

## Results

### Generation and characterization of 34 HPV PsV types

PsV are useful surrogates for biologic studies of native virions, particularly given their capacity to recapitulate early events of papillomavirus infection and neutralization, deliver a reporter gene of choice and their relative ease of production [39]. Here we encapsidated a vector expressing firefly luciferase (pY-luc) into HPV PsV because of this reporter's low background, high sensitivity and utility in the mouse challenge model [35]. Recombinant mammalian expression of papillomavirus capsid genes is dramatically enhanced by their codon modification [40], [41], presumably reflecting the disruption of negative regulatory sequences [42]. Several PsV vectors have been previously described [39], [43], [44], [45], [46] and we generated constructs for additional HPV types that were not previously available (Sequences in Table S1 and constructs may be obtained from Addgene (www.addgene.com)). HPV PsV were prepared by co-transfection of 293TT cells with pY-luc and the L1/L2 expression vectors per standard methodology [39].

**Figure 1:**
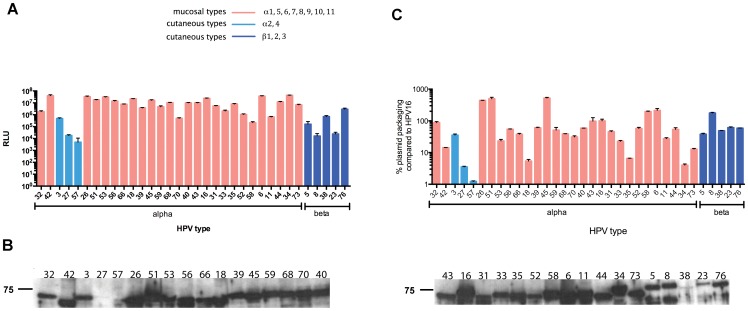
Assessment of infectivity, L2 incorporation, and reporter DNA encapsidation by PsV preparations of 34 HPV types. (A) 250 million particles (as estimated based on L1 concentration) of HPV PsV were transferred to 293TT cells, incubated at 37°C for 72 hours before cell lysis. Luciferase activity was measured in cell lysate (n = 4). (B) Western blot analysis detecting L2 in 100 ng of each HPV PsV preparation using rabbit antiserum to L2 α11-88x8. (C) Reporter plasmid copy number of each PsV preparations for each HPV type sample was measured by quantitative real time PCR. Black and blue bars represent alpha and beta type, respectively, and mean ± standard error plotted.

We first tested infectivity of each HPV PsV type normalized to 250 million particles (as estimated based on L1 concentration) by measuring luciferase activity (relative light units, RLU) 72 h post-infection in 293TT cells. The HPV PsV infected cells showed dramatic differences in reporter expression (Figure 1A). Overall, the cutaneous HPV type PsV showed lower infectivity compared to mucosal tropic types (p<0.0001), and the PsVs derived from alpha species HPV showed greater infectivity that those derived from beta HPV types (p = 0.01). However, even for the weakest PsV types, the signal observed was still more than 100-fold higher than background signal. Furthermore, pre-incubation of the PsV with rabbit antiserum raised against the cognate L1 VLP prevented reporter delivery, consistent with antibody-mediated neutralization of an infection rather than a non-specific transfection. Rabbit antiserum to L1 VLP was not available for a number of HPV types. Instead, sera was harvested from mice two weeks after electroporation i.m. three times at two week intervals with the an HPV L1 expression vector [47]. Pre-incubation of the HPV PsV with these mouse antisera similarly inhibited reporter transfer (Figure S1).

It is possible that the weak infectivity of the PsV derived from cutaneous HPV types reflects use of 293TT cells a target cell line, rather than one derived from skin cells. Therefore we also tested infectivity of diverse alpha and beta HPV PsV in HeLaT clone 4 cells, a human cervical cancer line transduced with SV40 large T antigen [48], as well as in the spontaneous immortalized human keratinocyte cell line HaCaT [49], and KH-SV, a human keratinocyte line immortalized by transduction with SV40 large T antigen [50]. In both human skin keratinocyte-derived lines (HaCaT and KH-SV), the cutaneous HPV PsV demonstrated minimal or undetectable signals, whereas mucosal HPV PsV showed more robust infectivity. Similar findings were seen for infection of HeLaT cells, implying that the weaker infectivity of the cutaneous HPV PsV does not reflect the use of 293TT as target cells or the need to infect a line derived from skin keratinocytes (Figure S2A, B, C).

L2 is known to play several important functions during virion assembly and infection, and therefore differences in the incorporation of L2 may account for the wide range of PsV infectivity by HPV type. Thus, the level of L2 incorporation into the gradient-purified preparations of HPV PsV was analyzed by Western blot (Figure 1B) using a broadly reactive rabbit antiserum to the L2 antigen α11-88x8 [35]. This Western blot analysis suggested that the PsV preparations of several cutaneous HPV types that exhibited low infectivity (Table 1) also contained low levels of L2, notably HPV23, 27, 38 and 57 (Figure 1B). This does reflect the failure of the antiserum to recognize the L2 of these HPV types because this antiserum, but not the pre-immune serum, can neutralize their infection. Furthermore, the L2 band was not apparent on Coomassie-stained SDS-PAGE analysis of these PsV. Rather, this reflects poor expression of L2 and thus low incorporation into the PsV, as Western blot analysis of 293TT cells transfected with these constructs shows low levels of L2 (data not shown).

**Table 1:** Summary of the phylogeny, tropism, charge and in vitro neutralization by L2 11-88x8 antiserum of the 34 HPV genotypes tested.

Subfamily	HPV type	Mucosal/Cutaneous	Predicted net charge at pH 7.4	Neutralization titer for L2x8 antiserum	95% confidence interval in neutralization titer
α1	32	M	5.2	3,360	2,225 to 5,074
	42	M	3.4	11,450	9,456 to 13,865
α2	3	C	2	11,140	7,314 to 16,967
α4	27	C	−4.2	3,942	350 to 44,402
	57	C	1	3,440	858 to 13,789
α5	26	M	5.2	11,223	7,786 to 16,177
	51	M	5.2	4,420	3,755 to 5,202
α6	53	M	6.3	352	208 to 595
	56	M	5.2	1,536	1,243 to 1,898
	66	M	7.2	870	517 to 1,465
α7	18	M	5.9	12,422	10,780 to 14,312
	39	M	5.1	6,216	3,798 to 10,174
	45	M	5.9	4,913	3,021 to 7,991
	59	M	7.1	8,214	7,255 to 9,300
	68	M	3.1	1,403	980 to 2,008
	70	M	4.1	2,359	1,382 to 4,027
α8	40	M/C	4.2	834	380 to 1,830
	43	M/C	6.2	1,580	1,063 to 2,349
α9	16	M	2	31,266	28,513 to 34,284
	31	M	8	2,212	940 to 5,207
	33	M	4.8	16,207	9,942 to 26,420
	35	M	7	7,458	5,151 to 10,800
	52	M	5.9	927	651 to 1321
	58	M	4.5	14,807	8,904 to 24,622
α10	6	M	7.1	5,553	4,315 to 7,146
	11	M	7.2	27,760	23,455 to 32,855
	44	M	7	5,041	2,716 to 9,358
α11	34	M	5.7	13,140	11,689 to 14,770
	73	M	4.9	8,810	6,853 to 11,325
β1	5	C	−4.7	3,561	492 to 25,770
	8	C	−2.8	17,679	7,256 to 43,073
β2	38	C	−4.8	1709	489 to 5976
	23	C	−3.9	2475	834 to 7347
β3	76	C	−4.9	2110	1154 to 3861

Phylogeny and tropism were taken from de Villiers et al [58], predicted net charge of L1 at pH7.4 was calculated as described in [37]. PsVs of each indicated HPV type carrying a luciferase reporter gene were mixed with titrated rabbit L2 α11-88x8 antiserum for two hours at 37°C, then the mixtures were transferred to 293TT cells and cultured for 72 hours. Cells were then lysed and luciferase activity was measured. Neutralization titer and 95% confidence interval are shown.

Differences in the infectivity of each type of HPV PsV might also reflect variable efficiency in reporter plasmid encapsidation. Therefore the number of copies of benzonase-resistant, and therefore presumably encapsidated, reporter plasmid DNA present within 315 ng of each gradient purified HPV PsV preparation was measured by quantitative real time PCR. The lowest levels of reporter DNA encapsidation were observed for PsV of HPV27, 57, and 34 (Figure 1C). Since L2 has been shown to facilitate genomic DNA encapsidation, the low DNA encapsidation by HPV types 27 and 57 may reflect the low levels of L2 incorporated in these PsV. However the amounts of encapsidated plasmid and/or L2 were not always proportional to the relative infectivity (Figure 1A,C), as seen for HPV18 and HPV35 PsV.

### Inhibition of HPV infection by Heparin and I-carrageenan

**Figure 2:**
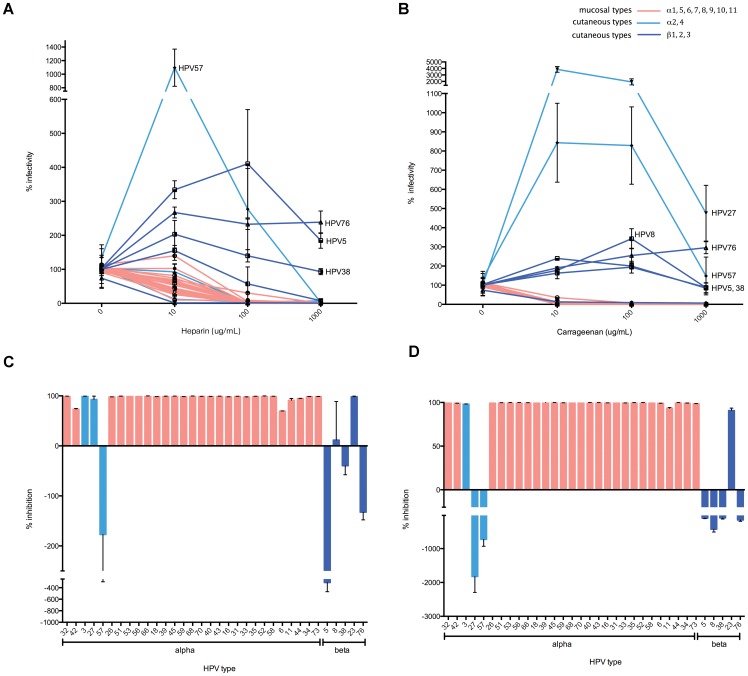
Inhibition of HPV PsV infection by titrations of heparin and carrageenan. PsVs of each HPV type carrying a luciferase reporter gene were incubated with 1000 µg/mL, 100 µg/mL, 10 µg/ml or 0 µg/ml heparin (A) or carrageenan (B) at 37°C for 1 hour. The mixtures were transferred to 293TT cells for 72 h. After incubation cells were lysed and luciferase activity was measured, and percent infection compared to control was calculated (n = 3). The percent inhibition of infection by each HPV PsV in the presence of 100 µg/mL of heparin (C) or carrageenan (D) was also plotted separately. Red and blue bars and lines represent mucosal and cutaneous HPV types, respectively.

CHO cells deficient in heparan sulfate proteoglycan (HSPG) production are no longer infected by HPV PsV [10], suggesting that HSPG is an important entry factor. Further soluble heparan sulfate can bind to capsids and is an inhibitor of infection for at least some HPV genotypes, but not all [14]. To explore the generalizability of these observations, we tested if heparin universally inhibited infection by PsV of all 34 HPV types within the alpha and beta HPV subfamilies. Ten-fold serial dilutions of heparin (Sigma H-4784), ranging from 10 to 1000 µg/mL, were incubated with the PsV of each HPV type for 1 hour at 37°C prior to transfer to 293TT cells to assess infectivity. Near complete inhibition was observed for PsV of all 29 alpha HPV types at 1000 µg/ml heparin, including HPV 31 (Figure 2A) for which the role of HSPG in infection is controversial [51]. With the exceptions of HPV8 and HPV23, infection by PsV of the beta HPV types was not impeded even at the highest concentration of heparin tested (1000 µg/mL), and paradoxically several demonstrated increased levels of infectivity [52]. Notably, the addition of 10 or 100 µg/mL heparin profoundly increased infectivity of HPV5, HPV57 and HPV76 PsV, slightly increased the infectivity of HPV38 PsV, but inhibited HPV8 and HPV23 PsV (Figures 2A and C). In contrast, even at 10 µg/mL heparin, most alpha HPV type PsVs exhibited significant inhibition.

Carrageenan is also a potent inhibitor of HPV infection via a mechanism resembling that of heparan sulfate [12], [13], [14], [15], although the breadth of its activity has been questioned recently [38]. It is currently being tested as a vaginal microbicide, and may be broadly protective against genital HPV transmission [17]. Therefore, we also tested the impact of carrageenan on in vitro infectivity of PsVs of diverse HPV types. The findings resembled the inhibitory profile of heparin [14], [37], [53]. PsV of most alpha types were inhibited except HPV27 and HPV57, but carrageenan had limited impact on beta types (Figure 2B, D). Carrageenan was more potent in inhibiting alpha HPV PsV infection than heparin at 10 µg/mL (Figure 2), consistent with earlier findings [12]. Carrageenan also showed some differences in inhibitory efficacy against PsV of beta HPV types compared to heparin. For example, carrageenan did not inhibit HPV27 infection whereas heparin did, suggesting subtly distinct mechanisms of inhibition by carrageenan and heparin. Taken together, the results imply that different HPV subfamilies bind HSPG differently during infection, which may contribute to their differing tropism [14]. Further, heparin and carrageenan are broadly inhibitory against PsV of most alpha species of HPV (including all of the high-risk mucosal HPV types tested), but not against PsV infection by beta or certain benign alpha types that can also infect cutaneous sites.

### Conservation of furin cleavage as an essential step for infection

**Figure 3:**
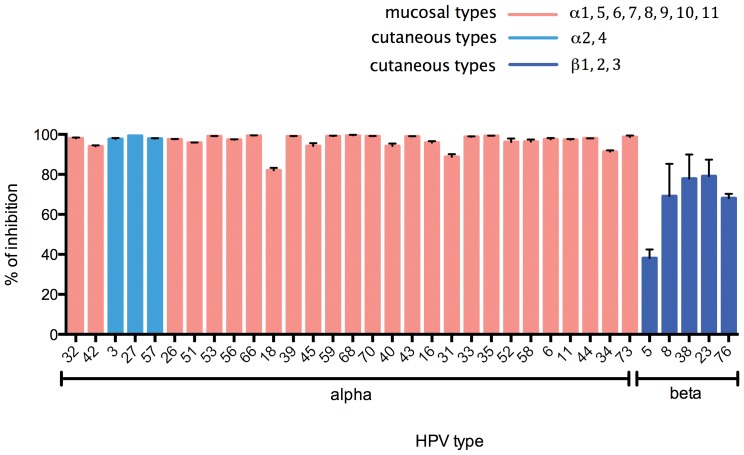
Impact of furin inhibitor on infection by PsV of diverse HPV. PsVs of each indicated HPV type carrying a luciferase reporter gene were transferred to 293TT cells for 72 hours in the presence or absence of 20 µM furin inhibitor. After incubation, cells were lysed, luciferase activity was measured and percent inhibition of infectivity compared to control calculated. Red and blue bars represent mucosal and cutaneous HPV types, respectively.

After binding to HSPG on extracellular matrix, HPV16 undergoes a conformational change and exposes the previously buried amino terminus of L2 on the capsid surface prior to cell entry [20], [21]. The exposed amino-terminus of L2 is sensitive to furin cleavage as it contains a conserved consensus cleavage site, Arg-X-(Arg/Lys)-Arg, and mutation of this site prevents HPV16 PsV infection. The furin inhibitor decanoyl-RVKR-cmk also can prevent HPV16 infection, suggesting cleavage of L2 by furin is a critical step for infection [20]. However, only a few HPV types have been tested and overall significance of furin cleavage among diverse HPV types has not been thoroughly addressed so far. Sequence alignment indicated that the furin target sequence, RX(R/K)R, is present in the L2 of all the HPV types for which we developed PsV, suggesting that the function provided by furin cleavage is conserved (data not shown). Therefore we tested whether application of the furin inhibitor at 20 µM (a concentration at which no cell toxicity was observed by XTT assay, data not shown) inhibited infection of 293TT cells by PsV of all 34 HPV types. Infection of 293TT cells by PsV of every alpha HPV was strongly inhibited, although it was less effective against some beta types such as HPV5 (Figure 3). Nevertheless, it is clear that the requirement for furin cleavage during PsV infection is ubiquitous among the 34 diverse HPV types tested.

### Conservation of need for cleavage by γ-secretase during infection

**Figure 4:**
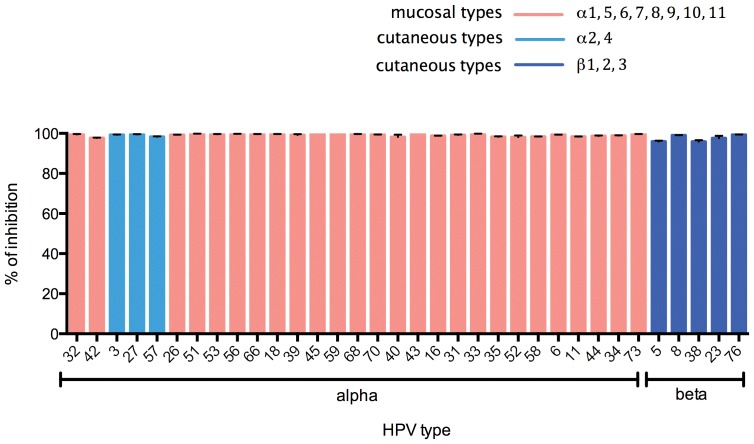
Impact of γ-secretase inhibitor on infection by PsV of diverse HPV. PsVs of each indicated HPV type carrying a luciferase reporter gene were transferred to 293TT cells for 72γ-secretase inhibitor XXI (n = 3). After incubation, luciferase activity was measured and percent inhibition of infectivity compared to control calculated. Red and blue bars represent mucosal and cutaneous HPV types, respectively.

The γ-secretase inhibitor XXI potently blocks HPV16 infection, and homozygous deletion of γ-secretase component subunits nicastrin or presenilin-1 also prevents HPV16 infection [27]. To determine whether the need for γ-secretase function during infection is conserved across diverse HPV types, we examined the impact of 500 nM XXI upon infection of 293TT cells by each PsV types. The γ-secretase inhibitor XXI dramatically reduced the infectivity of the PsV of all 34 HPV types tested (Figure 4), implying that γ-secretase is a key cellular factor that diverse genotypes utilize during infection and its inhibition can broadly inhibit HPV infection [27], [28].

### Neutralization of diverse HPV by antiserum to L2 antigen α11-88x8

The amino terminus of L2 contains a protective epitope and it is also evolutionarily highly conserved between types and even different species. We previously described that rabbit antiserum targeting the L2 antigen α11-88x8 can neutralize a broad spectrum of mucosal HPV types, notably those associated with cervical cancer and benign genital warts, and that passive transfer of this antiserum protects mice from genital HPV PsV challenge [35]. While this candidate vaccine antigen was designed to broadly protect against mucosal HPV that are associated with cervical cancer and genital warts, it may also protect against cutaneous HPV types as there is considerable sequence conservation in the 11-88 region. Cutaneous HPV types have been associated with non-melanoma skin cancers and benign skin warts that are costly to treat or a common nuisance, particularly for immunocompromised patients [6]. To examine the breadth of cross-neutralization against diverse HPV types, we tested antiserum from a rabbit vaccinated with the L2 antigen α11-88x8. PsV from each of the 34 HPV types was incubated with 2-fold serially diluted rabbit serum and the mixture was then transferred to 293TT cells to assess infectivity. The α11-88x8 antiserum detectably neutralized PsV of all alpha and beta HPV types tested (Table 1), whereas the pre-immune serum did not. Likewise, this antiserum reacted with L2 in PsV preprations of all 34 HPV types tested in a Western blot analysis (Figure 1B). These observations suggest the potential of this candidate vaccine antigen to protect against diverse beta types associated with non-melanoma skin cancer in immunocompromised and EDV patients.

## Discussion

The two licensed vaccines safely provide robust protection for men and women against acquiring infections by the two most potent oncogenic types, HPV16 and HPV18, and Gardasil also protects against the two most common types associated with genital warts, HPV6 and HPV11. In spite of their effectiveness, there is still a demand for new approaches for the prevention of HPV infection due to the high cost and type-restricted protection afforded by the licensed vaccines and also the need for immunization [54]. Aside from HPV16 and HPV18, about a dozen other oncogenic alpha HPV types are associated with cervical cancer, although each contributes only a minor fraction. Although the licensed vaccines provide varying degrees of cross-protection against closely related oncogenic HPV types, the extent and duration of cross-protection remain controversial [33]. Consequently, there are ongoing efforts to develop a nonavalent L1 VLP vaccine to target the seven oncogenic HPV types most commonly detected in cervical cancer, as well as HPV6 and HPV11. However, inclusion of 9 HPV VLP types into a single vaccine formulation will most likely increase the cost of vaccine production, which is one of the main limitations to global implementation of HPV immunization. Moreover, there has been little focus on vaccination against cutaneous HPV types, notably the beta species. While the beta HPVs typically have minimal clinical impact among patients with intact immune systems, immunocompromised patients (i.e. HIV-positive patients or those receiving immunosuppressive treatment following solid organ transplantation) and those with the rare hereditary syndrome EDV are vulnerable to non-melanoma skin cancers caused by these beta types, notably HPV5 and HPV8 [6]. It is unlikely that the licensed vaccines or even the new nonavalent vaccine will impact infection by the diverse beta HPV types, and therefore there is interest in alternative approaches to provide coverage against beta HPV.

More than 120 HPV genotypes has been completely characterized so far, but studies of the mechanisms of infection have been focused only on a very limited number of HPV types of the greatest medical significance, mainly due to restricted availability of PsV of a few HPV types. Therefore to assist in understanding the biology of HPV as well as assessing the breadth of protection provided by new vaccine and microbicide candidates, we generated and tested a diverse set of 34 PsV including 29 alpha types and 5 beta types (Table 1). The mechanisms underlying the different tropism of HPV are not understood. The alpha HPV types, with the exception of alpha2 and alpha4 subgroups, preferentially infect the genital mucosa and are sexually transmitted, whereas the beta HPV types infect the keratinizing squamous epithelium of the skin, typically starting during childhood. The distinct tropism may be governed by differences in entry into the cell, or viral transcription or replication, or a combination of these functions. To initially examine whether there are differences in the uptake processes of the predominantly mucosatropic alpha and cutaneotropic beta HPVs we tested the potency of known inhibitors of infection against PsV of the diverse range of HPV types. Use of PsV delivering a luciferase reporter for these studies eliminates any influence of viral tropism that would be conferred by the native viral genome with respect to viral transcription or replication.

The initial studies focused on HSPG because it is the most studied cell surface receptor for HPV, and there is evidence of differential utilization by different genotypes [14], [37], [38], [51]. We verified that the two HSPG mimetics heparin and carrageenan are inhibitors of diverse mucosatropic HPV PsV infection. This is significant because of ongoing efforts to develop carrageenan as a vaginal microbicide and its use as an ingredient in many sexual lubricants. Both compounds completely inhibit infection by most alpha types at high and intermediate concentrations (Figure 2), although most alpha types demonstrated partial inhibition by 10 µg/mL heparin, the lowest concentration tested (Figures 2A and C). However, while infection by HPV27 was blocked by heparin at 1000 µg/mL, carrageenan did not inhibit even at the highest concentration of 1000 µg/mL (Figure 2B), possibly reflecting their different structures and mechanism of particle binding [37], [53]. Notably, the alpha4 viruses HPV27 and HPV57 are capable of infecting cutaneous sites. A previous study suggested that cutaneous types tended to have negatively charged L1 at pH 7.4 and lower surface net charge compared to mucosal types [37]. We calculated a net charge for L1 of 34 HPV types at pH 7.4 and beta types (Table 1). Interestingly HPV27 and HPV57 L1 display the lowest net charge at pH 7.4 among the 29 alpha types and they were not inhibited effectively by carrageenan at a concentration of 1000 µg/mL, 100 µg/mL and 10 µg/mL like most beta types and HPV57 was not inhibited by heparin at 100 µg/mL and 10 µg/mL. These net charge differences may contribute to the distinct response of HPV27 and 57 PsV to the HSPG mimetics as compared with the other alpha types [37], [53].

When the concentration of heparin or carrageenan was reduced to 10 µg/mL, PsV of some cutaneous HPV types demonstrated enhanced infectivity (Figure 2A, B). This result was unexpected because heparin and carrageenan are inhibitors of mucosatropic HPV infection, although low amounts of heparin increased HPV infectivity in a recent report [52], [55], [56]. It is possible that when virion surface is partially coated by heparin or carrageenan, it can trigger a conformational change which, in turn, enhances infectivity perhaps by rendering L2 more accessible to furin cleavage [52]. Conversely, if the virion surface is completely coated by heparin or carrageenan due to high affinity, and if the binding groove and charged residues on the capsid surface that interact with the HSPG cellular receptor are masked completely [10], this can potentially prevent cell binding and infection.

Despite efficiently blocking infection by the mucosatropic alpha types, neither heparin nor carrageenan were consistent inhibitors of the cutaneous types, and indeed frequently promoted infection. This failure of heparin to inhibit infection by one beta type was also observed previously for HPV5 [56]. Several studies suggest that key surface exposed positively charged lysine residues in L1 are important for interaction of HPV16 PsV with heparan sulfate, and mutation of these lysine residues resulted in reduced cell binding and infectivity. We aligned L1 of 34 HPV types tested and noted only one of these lysine residues, HPV16 L1 278K, is highly conserved in alpha types but not beta types (data not shown). Differences in interaction with HSPG-like molecules on the cell surface may account in part for the tropism of HPVs for cutaneous versus mucosal epithelia [14]. However, the relationship is not straight forward; heparin inhibited HPV3, HPV23 and HPV27, and carrageenan inhibited HPV3 and HPV23 infection (Figure 2). It is unclear whether heparin and carrageenan fail to inhibit most beta HPVs because they simply cannot bind to the virion, or they do bind but cannot block receptor interaction. A failure to bind beta HPV particles is unlikely since many PsV showed increased infectivity with addition of heparin and carrageenan. A potential concern with these observations is that low numbers of infectious particles in the PsV preparation of the cutaneous HPV types could have led to an artifact, although L2 incorporation and encapsidation of reporter plasmid was similar to the alpha types. It is also important to recognize considerable variability in L2 incorporation, genome encapsidation and infectivity for PsV preparations of different HPV types when using them as a model for native virions assembled in warts [38].

We expected to observe broad spectrum blockade of HPV infection by furin inhibitor because the furin cleavage consensus sequence RX(R/K)R in L2 was conserved for all HPV types examined herein. This sequence and the requirement for furin cleavage is also conserved in bovine (BPV) and other animal papillomaviruses [20]. Furin inhibitor blocked infection by all HPV types tested although not completely for some beta PsV. This may reflect use by the beta types of protein convertases other than furin that are partially inhibited by furin inhibitor [20], or possibly an artifact relating to the lower signal to noise ratio for the less infectious types.

The γ-secretase inhibitor XXI also effectively inhibited PsV infection for all HPV types tested (Figure 4). Beta types, which tend to exhibit somewhat lower sensitivity than alpha types to the furin inhibitor, were strongly inhibited by XXI. The data suggest that all the HPVs follow a common entry pathway dependent upon both furin- and γ-secretase-dependent proteolysis, despite potential differences in interactions with cell surface HSPGs between the mucosal and cutaneous types. However, while several γ-secretase inhibitors that have been tested clinically for prevention of Alzheimer disease might be re-tasked for broad prevention of HPV infection, they have also been associated with squamous cell cancer [57].

Although the amino terminus of L2 is a weaker immunogen compared to L1 VLP, it is considered to provide broader protection [29]. In particular, the concatenated fusion protein L2 α11-88x8 comprising residues ∼11-88 of L2 derived from eight alpha HPV types [35] has been shown to induce cross-neutralizing antibodies that broadly protect against the HPV types most commonly found in cervical cancer and benign genital warts. Here we also examined L2 α11-88x8 antiserum for its capacity to cross-neutralize cutaneous beta HPVs that are also associated with cancer, particularly among immunocompromised and EDV patients. Remarkably, the L2 α11-88x8 antiserum neutralized all beta HPV types tested (Table 1). The result implies that the neutralizing epitopes at the amino terminus of L2 are highly conserved and play an important role in both alpha and beta HPV infection, and that an L2 α11-88x8 vaccine might potentially provide broad protection against the diverse alpha and beta HPV family. Serologic studies suggest that beta HPV infections are acquired throughout life, beginning in childhood [6]. Therefore, optimally vaccination against beta HPV would need to begin early.

In conclusion, we have considered agents that block infection via inhibiting receptor interaction, furin cleavage or γ-secretase function as alternative approaches to vaccination to broadly prevent both mucosal and cutaneous HPV infections. At present, carrageenan shows promise as a vaginal microbicide to broadly prevent infection by mucosatropic high-risk papillomaviruses [17]. However our results show that it is unlikely to prevent cutaneous HPV transmission. Further, it would need to be regularly applied at high amounts to ensure full protection. As both furin and γ-secretase contribute to numerous cellular processes, systemic use of their inhibitors are also likely to have significant side effects rendering them impractical despite broad anti-HPV activity, although topical application might be feasible. Nevertheless, our study with 34 medically significant HPV types indicates that there is a conserved infection pathway once the virus enters its host cell, and thus targeting conserved residues of the capsid proteins that interact with the key cellular entry factors has potential for broad protection. Indeed, rabbit antisera to the L2 α11-88x8 antigen provided broad protection against alpha HPV challenge via cross-neutralizing antibodies [35]. Here we find that L2 α11-88x8-specific antibodies also cross-neutralize diverse beta papillomaviruses, suggesting the potential to protect against types associated with non-melanoma skin cancer in immunocompromised or EDV patients (should they mount a sufficient response after vaccination) or the potential of herd immunity in the general population to limit their circulation. Taken together, this study supports the possibility of vaccination with L2 candidate vaccines for pan-prevention of HPV infection.

## Supporting Information

Figure S1 **Neutralization of PsV by L1 VLP antiserum.** PsVs of each indicated HPV type, each carrying a luciferase reporter gene, were mixed with mouse or rabbit L1 VLP antiserum or their respective pre-immune serum (each at 1∶50 dilution) for two hours at 37°C, then the mixtures were transferred to 293TT cells and cultured for 72 hours (n = 3). Cells were then lysed and luciferase activity was measured. Percent neutralization by L1 VLP antiserum was plotted. Pre-immune serum was non-neutralizing in all cases. Red and blue bars represent mucosal and cutaneous HPV types, respectively.
(TIF)

Figure S2 **Infectivity of diverse HPV on mucosal and skin cell lines.** HeLaT (A), HaCaT (B), KH-SV (C) cells were treated with titrations of diverse HPV PsV from alpha and beta subfamilies and incubated at 37°C for 72 hours. Cells were lysed after incubation and luciferase activity was measured. Red and blue bars represent mucosal and cutaneous HPV types, respectively.
(TIF)

Table S1 **Sequences of codon modified L1 and L2 genes utilized in expression constructs used to generate new HPV PsV types.**
(XLSX)
