# Supplementary figures and images for: Impact of Inhibitors and L2 Antibodies upon the Infectivity of Diverse Alpha and Beta Human Papillomavirus Types
Source: PLoS One. 2014 May 9;9(5):e97232. doi: 10.1371/journal.pone.0097232 (PMC4016295; doi:10.1371/journal.pone.0097232)

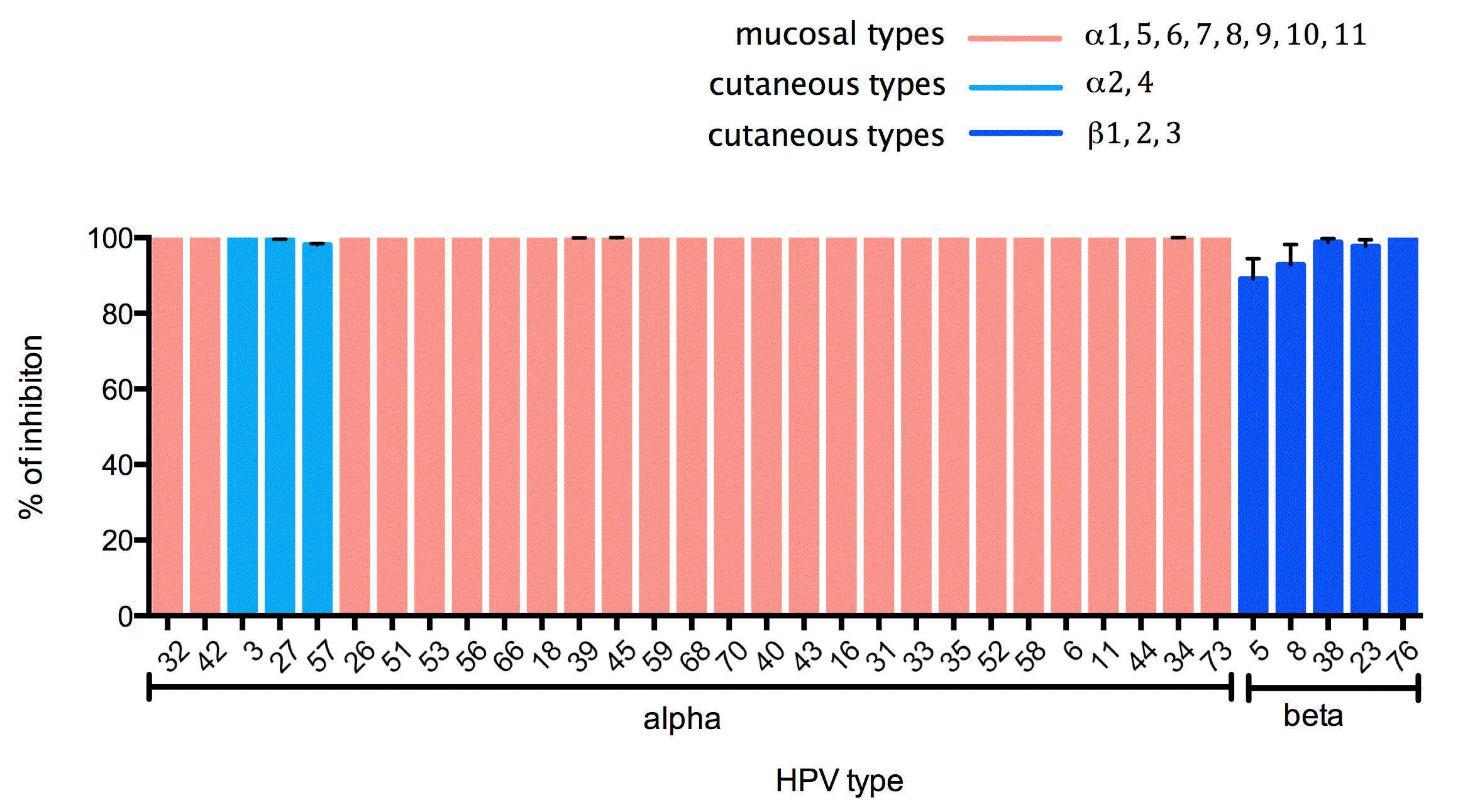

Supplement: Figure S1 — Neutralization of PsV by L1 VLP antiserum. PsVs of each indicated HPV type, each carrying a luciferase reporter gene, were mixed with mouse or rabbit L1 VLP antiserum or their respective pre-immune serum (each at 1∶50 dilution) for two hours at 37°C, then the mixtures were transferred to 293TT cells and cultured for 72 hours (n = 3). Cells were then lysed and luciferase activity was measured. Percent neutralization by L1 VLP antiserum was plotted. Pre-immune serum was non-neutralizing in all cases. Red and blue bars represent mucosal and cutaneous HPV types, respectively. (TIF) [file pone.0097232.s001.tif]

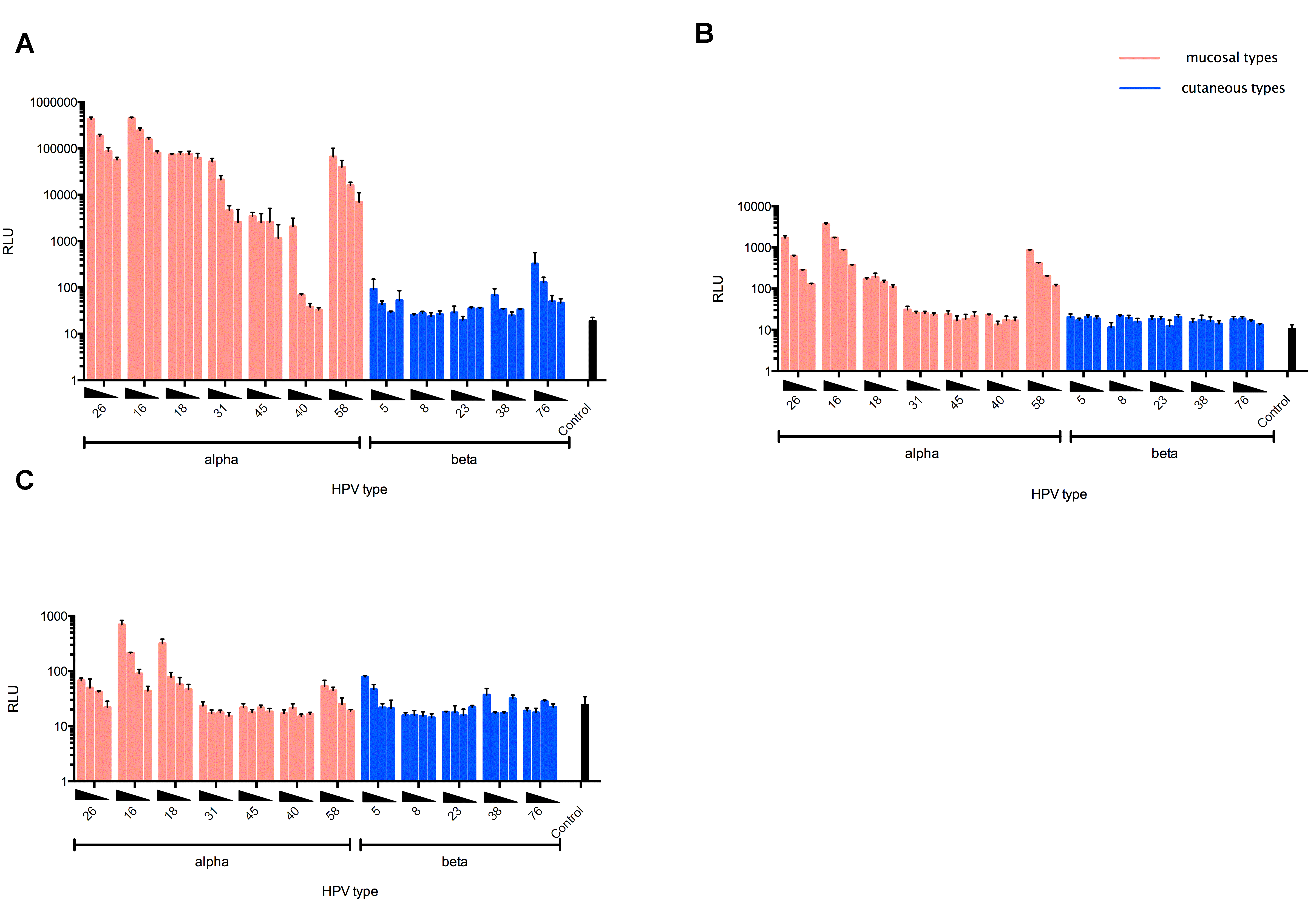

Supplement: Figure S2 — Infectivity of diverse HPV on mucosal and skin cell lines. HeLaT (A), HaCaT (B), KH-SV (C) cells were treated with titrations of diverse HPV PsV from alpha and beta subfamilies and incubated at 37°C for 72 hours. Cells were lysed after incubation and luciferase activity was measured. Red and blue bars represent mucosal and cutaneous HPV types, respectively. (TIF) [file pone.0097232.s002.tif]
